# Family Systems and Emotional Functioning in Deaf or Hard-of-Hearing Preschool Children

**DOI:** 10.1093/deafed/enab044

**Published:** 2022-01-31

**Authors:** Shannon Yuen, Boya Li, Yung-Ting Tsou, Qi Meng, Liyan Wang, Wei Liang, Carolien Rieffe

**Affiliations:** Unit of Developmental and Educational Psychology, Institute of Psychology, Faculty of Social and Behavioral Sciences, Leiden University; Unit of Developmental and Educational Psychology, Institute of Psychology, Faculty of Social and Behavioral Sciences, Leiden University; Unit of Developmental and Educational Psychology, Institute of Psychology, Faculty of Social and Behavioral Sciences, Leiden University; Unit of Developmental and Educational Psychology, Institute of Psychology, Faculty of Social and Behavioral Sciences, Leiden University; China Rehabilitation Research Center for Hearing and Speech Impairment; China Rehabilitation Research Center for Hearing and Speech Impairment; Unit of Developmental and Educational Psychology, Institute of Psychology, Faculty of Social and Behavioral Sciences, Leiden University; Department of Human Media Interaction, Faculty of Electrical Engineering, Mathematics and Computer Science, University of Twente; Department of Psychology and Human Development, Institute of Education, University College London

## Abstract

This study examined how deaf or hard-of-hearing (DHH) and typically hearing (TH) children may differ in their family system and emotional functioning and examined the relations between family system and children’s emotional functioning. Parents of 106 DHH and 99 TH children (2–6 years) reported on family cohesion and adaptability, parental emotion communication, and their child’s emotional functioning. The DHH children were rated lower on family cohesion and positive emotion expression than the TH children. Higher levels of family cohesion related to more positive emotion expression in TH children but not in DHH children. For all children, higher levels of family cohesion related to fewer negative emotion expressions and more parental emotion communication related to more negative emotion expression. The results emphasize the importance of sharing leisure activities together and open communication within the family, which can support DHH and TH children’s experience of emotions and their expressions of them.

The family system is essential for young children’s emotional development. From the first day of a child’s life onward, family members’ interactions with each other set up examples and provide crucial learning opportunities for children to develop the skills to understand others’ emotions and to express their emotions in a socially acceptable and adaptive way (Hosie et al., 2000; Ketelaar et al., 2017; Suveg et al., 2014). However, the quality and quantity of family interactions could be rather different in families with a deaf or hard-of-hearing (DHH) child, compared to families with a typically hearing (TH) child, which in turn could influence DHH children’s emotional development. The majority of DHH children are born to TH parents and grow up in a hearing environment surrounded by spoken language from the home to school (Mitchell & Karchmer, 2004). Before their DHH child was born, many hearing parents had no prior experience interacting closely with DHH individuals. This lack, combined with the unexpected hearing differences of their child, can raise difficulties and stress within the family context, especially in the first couple of years after the DHH child is born (Calderon & Greenberg, 2011; Feher-Prout, 1996; Ketelaar et al., 2017; Koester & Lahti-Harper, 2010; Necula et al., 2018; Spahn et al., 2003; Vaccari & Marschark, 1997; Weisel et al., 2007; Zaidman-Zait et al., 2016). On the other hand, having to deal with a challenging situation can make family members feel more united and prompt them to develop coping strategies and resilience against stressors (Ahlert & Greeff, 2012; Jackson et al., 2010). No doubt, raising a DHH child in a hearing family will change the dynamics and the system of the family. However, it remains largely unknown to what extent the family system will be altered by having a DHH child in the family where other family members are hearing and how this would in turn influence DHH children’s emotional development. This study aimed to address the gap by investigating the family system and its relation to emotional functioning in preschool DHH children and their hearing families in comparison to TH children and families.

The family system consists of interconnected family members where every action, emotion, and interaction can impact the other (Cox & Paley, 2003) and thus altogether depicts the level of functioning of the family. The conceptualization of the family system describes (1) cohesion, (2) adaptability, and (3) communication as the three core components central to defining family interactions (Kouneski, 2000; Olson, 2000; Olson et al., 1982).

*Family cohesion* refers to the level of emotional bonding between the family members (Olson, 2000). In a coherent family system, family members are emotionally connected to each other, have shared interests, and enjoy spending qualitative time together. To the best of our knowledge, only two studies have compared the level of family cohesion between hearing families with DHH children and families with TH children and found higher levels of family cohesion in the families with a DHH child (Holt et al., 2013; Necula et al., 2018). Spending quality time together and participating in shared activities fosters a stronger emotional bond within families. Past research found that families with children with physical or sensory disabilities usually had parents highly involved in their children’s lives and activities (Holt et al., 2013; Koester & Lahti-Harper, 2010; Luckner & Velaski, 2004; Pinquart, 2013; Spahn et al., 2003; Weisel et al., 2007; Zaidman-Zait et al., 2018). For hearing families with a DHH child, in addition to the daily routine, hearing parents of DHH children need to invest extra time on the access to communication in the family environment, especially during their participation in early intervention programs (Jackson & Turnbull, 2004; Necula et al., 2018). While for TH children and their parents, leisure activities provide an important channel for spending quality time together, the engagements in treatment and intervention programs may constitute a large portion of the quality time spent by DHH children and their parents (Jackson et al., 2010). Despite the stress for some families, the shared expectations for better outcomes by parents and child and the undivided focus of parents on their child’s needs and well-being may contribute to stronger emotional bonding and parent–child synchrony with higher levels of family cohesion (Thomson et al., 2011).

*Family adaptability* refers to the family’s ability to involve each family member with participating in decision-making and problem-solving, and it allows everyone to be equally heard and to implement any change of rules within the family (Olson, 2000). A well-functioning family thus requires the ability to keep adapting to new changes and to fine-tune their strategies to meet each family member’s needs (Feher-Prout, 1996; Jackson et al., 2010; McCubbin, 1995; Zaidman-Zait et al., 2016). To date, insufficient studies have directly examined adaptability of families with a DHH child. Yet, indirect evidence suggests that hearing families with DHH children should be able to adapt well to the child’s disability, with some parents taking more time to explain family rules to their DHH child (Ahlert & Greeff, 2012; Antonopoulou et al., 2012; Holt et al., 2013; Jackson et al., 2010; Necula et al., 2018). On the one hand, the special situation that families with a DHH child face may encourage family members to adapt their rules, roles, and communication styles more frequently to meet their children’s special needs than families with TH children (Ahlert & Greeff, 2012; Zaidman-Zait et al., 2016). On the other hand, the constant demand of managing new stressors and making new changes could lead to confusion for parents of DHH children in which role to take within the family system and to self-doubt in their child-rearing abilities, leading to a lack of information for adequate decision-making (Ahlert & Greeff, 2012; Antonopoulou et al., 2012; Dirks & Rieffe, 2019; Feher-Prout, 1996; Zaidman-Zait et al., 2016). The frustration and confusion in parents can result in the enforcement of rigid discipline (stricter family rules and roles) in order to (re)gain control over the stress (Holt et al., 2013; Spahn et al., 2003), which may explain rigid and directive parenting style often observed in families with DHH children, as compared to families with TH children (Koester & Lahti-Harper, 2010; Necula et al., 2018; Pinquart, 2013).

*Emotion communication* in the family refers to their ability to listen to each other and to share and discuss emotions about themselves and about the relationship with each other (Dirks et al., 2020; Olson, 2000. This type of communication is often conducted through verbal conversations and requires complex linguistic abilities, and thus can be challenging for hearing families with DHH children (Barker et al., 2009; Calderon & Greenberg, 2011; Sidera et al., 2017; Vaccari & Marschark, 1997). Although, nowadays, most DHH children receive the help from hearing devices, many still experience difficulties in hearing, especially when the environment is noisy or when the speaking partner is far away or not facing toward the child (Koester & Lahti-Harper, 2010). To adjust to the hearing status of the child, families with a DHH child often spend less time in mutual exchanges in conversation and the conversations consist of simpler content that may not always describe accurately what one wishes to communicate (Calderon & Greenberg, 2011; Dirks & Rieffe, 2019; Jackson & Turnbull, 2004; Morgan et al., 2014; Vaccari & Marschark, 1997). For example, verbal communication can be exhausting for both the TH parent and the DHH child and therefore every episode of the conversation tends to be short. The parents need to resort to physical means of communication to direct and sustain the attention of their child, such as shoulder tapping or waving (Koester & Lahti-Harper, 2010; Vaccari & Marschark, 1997), and the DHH child, in addition to attending to the auditory input, also has to pay close visual attention and read the lips during verbal communication. Moreover, termed as “linguistic overprotection,” TH parents of DHH children often reduce the linguistic and cognitive complexity in conversations due to a mixture of fear of being misunderstood by their DHH child or of misunderstanding their child (Calderon & Greenberg, 2011; Dirks & Rieffe, 2019; Kluwin & Gaustad, 1994; Morgan et al., 2014).

## Relations Between the Family System and DHH children’s Emotional Functioning

An important developmental task for young children is to learn how to express their emotions in a socially accepted and adaptive way and how to correctly interpret others’ emotions to ensure successful social interactions (Hosie et al., 2000; Morris et al., 2007). These emotional skills develop through observing and participating in social interactions. A great deal of social interactions is facilitated by good hearing and speaking abilities. For example, overhearing adults talking about emotions and discussing emotional issues with parents and friends helps children to understand and cope with emotions. Many DHH children who were born into a hearing family do not have full access to social learning and seem to lag behind TH children in their emotional development (Buss & Kiel, 2004; Hosie et al., 2000). Although many preschool DHH children are seen to present similar levels of positive emotion expressions as TH children (Wiefferink et al., 2012), they express negative emotions more often, with more intensity than TH children, and were less able to divert their attention away from negative stimuli when provoked (Rieffe et al., 2003; Rieffe & Meerum Terwogt, 2006; Ziv et al., 2013). Aside from this, preschool DHH children who were born into a hearing family also take longer to develop their emotion recognition skills (Sidera et al., 2017; Wang et al., 2016) and are known to experience more difficulties in facial and vocal emotion recognition than TH children (Laugen et al., 2017; Most & Michaelis, 2012; Sidera et al., 2017; Wiefferink et al., 2013), although some studies still provide differing outcomes on this, and thus further research is needed to provide clarification (Jones et al., 2018; Most & Michaelis, 2012; Ziv et al., 2013).

The first social environment for children to learn essential emotional skills is the family (Necula et al., 2018), emphasizing the important role of parent–child interactions for emotional development (Castro et al., 2015; Cooke et al., 2016; Laugen et al., 2017; Morris et al., 2007; Sidera et al., 2017; Wiefferink et al., 2012). Expressing their emotions allows children to signal their intentions and desires (Scherer, 2000). However, cultural and social rules which are taught explicitly and implicitly within a family prescribe when and how to express one’s emotions to achieve this goal without aggravating the other person (Scherer, 2000; Suveg et al., 2014). In addition, it is also important for children to be able to read other people’s emotions and to understand the information conveyed by others’ emotion expressions, because when a child encounters a social situation, they must first appraise it and respond effectively to it. Likewise, currently, research on TH children has shown that parents need to be available to validate and support their child’s emotions in order to reduce their emotional arousal and so children can then regulate their expressions (Gao & Han, 2016; Panfile & Laible, 2012; Shaffer et al., 2012). Additionally, families can adapt to stressful situations with rigid family rules (i.e., family members should not express anger openly), which prompt their child to develop fewer coping strategies for negative emotions as they encounter less variations in emotions in the family and have not learnt the skills to regulate them (Necula et al., 2018).

Similarly, there is no indication that hearing families with a DHH child may express emotions differently to TH families even though parental stress has the potential to play a larger part in interrupting family interactions (Calderon & Greenberg, 2011; Dirks et al., 2016; Ketelaar et al., 2017; Koester & Lahti-Harper, 2010; Necula et al., 2018; Spahn et al., 2003; Zaidman-Zait et al., 2018;). Comparatively, studies have also found that TH children with stronger emotional bonding (i.e., cohesion; Cooke et al., 2016) and increasingly more discussions on labeling emotions between parent and child (communication) are reported to demonstrate better abilities in recognizing emotions (Castro et al., 2015; Cooke et al., 2016). While families with DHH children may have similar or even a stronger emotional bonding between family members, their difficulties in communicating with each other could hinder DHH children from developing their emotional skills at the same pace as their TH peers. Nonetheless, to the best of our knowledge, there is no literature examining the linkage between family cohesion, adaptability, and parental emotion communication to DHH children’s emotion expression and recognition.

## Present Study

For health clinicians in the field, it is key to understand how hearing families with a DHH child function, so they can provide the best care at the earliest stage. This is no simple task as recent studies have presented mixed findings on the quality of the family system for families with a DHH child. The aim of this study is to gain more insight into family systems with a DHH child and how this is related to the child’s emotional functioning. The particular focus of this study is on DHH children brought up in a hearing environment and surrounded by the spoken language. To our knowledge, studies that have looked at family components in relation to emotional components are limitedly available for DHH children, especially for preschool children. This study focused on children between 2 and 6 years of age because this is the stage at which children acquire the knowledge about basic emotions and predominantly learn from their nuclear family setting (Most & Michaelis, 2012).

The first aim of this study was to examine the extent to which family systems differ according to preschool children’s hearing status. We expected hearing families with a DHH child to show higher levels of family cohesion than families with a TH child (Holt et al., 2013; Necula et al., 2018) and lower levels of parental emotion communication (Calderon & Greenberg, 2011; Kluwin & Gaustad, 1994; Vaccari & Marschark, 1997). As to family adaptability, we did not make a specific hypothesis because there is a lack of studies in this area and circumstantial evidence gives inclination for both ways.

Second, we aimed to examine the relation between the family system and children’s emotional functioning. Already, previous research has given evidence that DHH children express more negative emotions than TH children, yet are similarly expressive of positive emotions. DHH children are also shown to lag behind TH children in emotion recognition (Most & Michaelis, 2012; Sidera et al., 2017; Wang et al., 2016; Wiefferink et al., 2013). Looking at the relation between family functioning and children’s emotional functioning, we expected better family cohesion, adaptability, and parental emotion communication to be related to more positive emotion expressions, fewer negative emotion expressions, and better emotion recognition in TH children. Due to the lack of empirical evidence, our hypotheses regarding DHH children were explorative in nature. We expected to find similar relationships in DHH children as in TH children.

## Methods

### Participants

A sample of 205 children (106 DHH, 99 TH; 123 boys, 82 girls) of ages 2–6 years (*M* = 5.25 months; standard deviation [*SD*] = 13.18) were recruited from a hearing and speech early intervention center (DHH sample) and from a kindergarten (TH sample) in China. The majority of the DHH sample had bimodal hearing devices and profound hearing loss. The DHH children in this sample live in a predominantly hearing environment, surrounded by the spoken language. All the DHH children attended special education classes embedded in their early intervention program, where they were all in classes with only DHH peers. The program consisted of speech and aural therapy. Sign language was not included in the education, as there was an emphasis placed on learning spoken language. There were also parent and child-focused classes provided for children <2.5 years of age. See Table 1 for participant characteristics.

**Table 1:** Characteristics of the sample

	DHH (*n* = 106)	TH (*n* = 99)
Personal characteristics		
Age, months, mean (*SD*)^***^	45.17 (12.54)	55.69 (11.64)
Gender, *n* (%)		
Male	61 (57.5)	62 (62.6)
Female	45 (42.5)	37 (37.4)
Nonverbal intelligence score,^b^ mean (*SD*)	0.1 (0.6)	0.2 (0.6)
Socioeconomic status, *n* (%)		
Maternal education		
Primary Secondary Tertiary Postgraduate Unknown Paternal education	3 (2.8) 35 (33.0) 59 (55.7) 7 (6.6) 2 (1.9)	2 (2.0) 31 (31.3) 33 (33.3) 4 (4.0) 29 (29.3)
Primary Secondary Tertiary Postgraduate Unknown Net household income	0 44 (41.5) 45 (42.5) 14 (13.2) 3 (2.8)	3 (3.0) 24 (24.2) 36 (36.4) 6 (6.1) 30 (30.3)
<€20,000 €20,000–€40,000 €40,000-65,000 €65,000–€130,000 >€130,000 Unknown	60 (56.6) 7 (6.6) 3 (2.8) 15 (14.1) 2 (1.9) 19 (17.9)	41 (41.4) 7 (7.0) 2 (2.0) 4 (4.0) 0 45 (45.5)
Hearing characteristics		
Age of Identification, months, mean (*SD*)	14.11 (13.86)	
Hearing device, *n* (%)		
CI (unilateral/bilateral)	2 (1.9)/14 (13.2)	
CI and HA	65 (61.3)	
HA only	18 (17.0)	
Others or unknown	7 (6.6)	
HA use, months, mean (*SD*)		
Age at HA fitting	23.8 (13.6)	
Duration of HA use	21.3 (12.1)	
CI use, months, mean (*SD*)		
Age at implantation	26.5 (12.4)	
Duration with (first) CI use	18.9 (10.7)	
Hearing threshold, *n* (%)		
Mild: 26–40 dB	0	
Moderate: 41–60 dB	10 (9.4)	
Severe: 61–80 dB	21 (19.8)	
Profound: >81 dB	68 (64.2)	
Unknown	7 (6.6)	
Preferred mode of communication, *n* (%)		
Spoken language only	92 (86.8)	
Sign-supported Chinese	12 (11.3)	
Sign language only	0	
Unknown	2 (1.9)	
Language score,^a^ mean (*SD*)		
Language production (age in months)	16.9 (13.4)	
Unknown, *n* (%)	27 (13.2)	
Language reception (age in months)	29.1 (19.2)	
Unknown, *n* (%)	33 (16.1)	

*Note.* DHH = deaf and hard-of-hearing; TH = typically hearing; HA = hearing aid; CI = cochlear implant; *SD* = standard deviation.

a All language scores of the DHH sample were corrected by age and reflect developmental stage (months).

b IQ scores were evaluated using different IQ test tools and were age-corrected and recoded based on their deviations from the grand population mean in the normative data: −2 = 2 *SD* below the mean; −1 = 1 *SD* below the mean; 0 = within 1 *SD*; 1 = 1 *SD* above the mean; 2 = 2 *SD* above the mean.

^***^ *p* < .001 between DHH and TH children.

The inclusion criteria for DHH children are: (1) deaf or hard of hearing before their third year of age, with a minimum of 40 dB HL in the best ear; (2) normal cognitive functioning (as indicated by teachers or medical doctors); and (3) no other additional disorder or disabilities. The TH group should meet the following criteria: (1) normal cognitive functioning (as indicated by teachers) and (2) without any disorder or disability. There were no significant differences found between groups on gender, household income, parent’s education level, and nonverbal intelligence scores, except for age, *t*(202.99) = 6.23, *p* < .001. The TH sample was on average older than the DHH sample.

To obtain an objective assessment of children’s cognitive functioning, nonverbal intelligence scores of DHH children were retrieved from the records of the early intervention center, where they were tested by either the Griffiths Mental Development Scales (Tso et al., 2017) or the Hiskey-Nebraska Test of Learning Aptitude (Yang et al., 2011). The TH children were tested by the researcher with the Wechsler Preschool and Primary Scale of Intelligence using the matrix reasoning, picture memory, and block design subtests (Li & Zhu, 2014). To allow for comparisons between different nonverbal intelligence tests, all raw scores were transformed into age-equivalent standard scores.

Initially, a total of 246 children and parents were approached. Yet, 20 children’s parents had not completed the questionnaires, 14 children were outside the age range of this study when the measures were administered to them, 5 children achieved nonverbal intelligence scores 2 *SD*s below the mean, and 2 children had DHH parents, leaving a final sample of 205 children in this study. The excluded sample did not differ from the included sample on age, gender distribution, hearing distribution, nonverbal intelligence scores, net household income, and parental education levels. Only the DHH group had language scores for production and reception, and no significant differences were found for the DHH children between the included and excluded samples.

## Materials

### Family System

#### Family adaptability and cohesion

The *Family Adaptability and Cohesion Evaluation Scale* (FACES-II; Olson et al., 1982) is a 30-item scale that assesses the family system in two dimensions: cohesion (16 items; e.g., “The relationship between family members is very close”) and adaptability (14 items; e.g., “When the family situation changes, the family’s normal life rules and house rules can easily change accordingly”). Family cohesion measures emotional connections, boundaries, time spent together, and shared interests and activities within the family. Family adaptability measures leadership, role relationships, and rules in a family (Olson et al., 1982). The FACES-II is an appropriate measure for use on a Chinese sample (Phillips, 1993). The FACES II was first translated and validated in China by Michael Phillips and his team in 1992, and they provided the norm scores on the Chinese population. Parents rated the items on a 5-point scale ranging from (1 = *almost never* to 5 = *almost always)*, and the scores were averaged for each scale; with higher scores indicating higher levels of cohesion and adaptability. Cronbach’s α of family cohesion subscale was .91, and for the family adaptability subscale, it was .86.

#### Parental emotion communication

The 10-item *Emotion Communication Questionnaire* (Wiefferink et al., 2015) was used in this study. Parents scored on their ability to discuss emotions with their children on a five-point scale on how often and in what depth they talked about emotions with their children (0 = *almost never*; 4 = *almost always*). An example item would be “I think it is important to teach my child to understand emotions.” Cronbach’s α was .79.

### Children’s Emotional Functioning

#### Emotional expression

The Negative Emotion Expression subscale (8 items) and Positive Emotion Expression subscale (6 items) were utilized from the *Emotion Expression Questionnaire* (*EEQ*, 35 items; Rieffe et al., 2010). On a five-point scale, parents scored the frequency, intensity, and duration of their child’s expressions of negative emotions, such as anger and sadness, and positive emotions of happiness and joy, as well as the extent to which the child can calm down from an emotional episode. Example items from both scales are “how often does your child show anger?” (1 = *almost never*; 5 = *almost always*) and “is your child easy to calm down when he/she is angry?” (1 = *very easy*; 5 = *very difficult*). Cronbach’s α of positive emotion expression subscale was .72, and for the negative emotion expression subscale, it was .76.

#### Emotion recognition

The 6-item *Emotion Recognition subscale* (*EEQ*, 35 items; Rieffe et al., 2010) from the *EEQ* was used in this study. Parents scored on their children’s ability to acknowledge their parent’s emotions and the extent to which they could understand their emotions on a five-point scale (1 = *almost never*; 5 = *almost always*). An example item would be “Does your child understand when you are angry?” Cronbach’s *α* was .77.

### Procedure

Prior to the data collection, the university’s ethics approval and parental informed consents were obtained. The EEQ and Emotion Communication Questionnaire were first translated by a bilingual translator from English to Chinese and was then back-translated to English to check for inconsistencies. Parent-report questionnaires were available in both paper and digital formats, and parents filled them out in one sitting. Additional variables, such as hearing history, socioeconomic status, and the living arrangements at home, were also on the questionnaire. DHH children’s nonverbal intelligence scores were collected from the records of the early intervention center. While, TH children were administered with WPPSI-IV subtests in a quiet room by the study researcher.

### Statistical Analyses

All statistical analyses were conducted on IBM SPSS 26.0 version. To address the first research question, we examined group differences (DHH vs. TH) in the family system and in the children’s emotional functioning using independent samples *t*-tests. To address the second research question, hierarchal regression analyses were performed to examine to what extent family cohesion and parental emotion communication explained children’s positive and negative emotion expressions and emotion recognition. To examine whether hearing status moderates these associations, interactions between family system variables and hearing status (coded as 0 = TH, 1 = DHH) were added to the regression analyses. All independent continuous variables were centered. Finally, to reduce bias caused by missing values and to address the age difference between DHH and TH groups, multiple imputation (MI) and weighting were conducted (detailed below), and pooled and weighted results are reported for all analyses.

### MI for Missing Values

Missing counts and percentage of missing of all study variables are presented in Supplementary Appendix S1 (Novin & Broekhof, 2019). Although, Little’s MCAR test showed a significant result thus data are not missing completely at random (χ*^2^* = 2548.58, *df* = 2,327, *p* = .001), given that the data were missing for known reasons, we assumed that data was missing at random (Azur et al., 2011; Novin & Broekhof, 2019). A thorough assessment of the pattern of the missing data (Supplementary Appendix S1) highlighted that some information on parental education per mother and father and net household income were not available. This could be largely due to the parents being unwilling to share their socioeconomic information (refer to Supplementary Appendix S1). Additionally, nonverbal intelligence scores were not available for 42 participants, either because the scores were not available from the early intervention center (33 DHH children), or children were absent from school due to illness, or were not tested due to time constraints (9 TH children). Missing data could mean a loss of power in the statistical analysis and a biased interpretation of the results, thus the MI technique was utilized. We created 10 imputation sets of all variables on SPSS (Graham, 2009), and control variables of age, hearing ability, gender, nonverbal intelligence test scores, net household income, and both parents’ education level were included in the estimation of imputed values along with all study variables (Azur et al., 2011). This technique thereby fills in missing values by assessing the characteristics of all participants.

### Weighting

There was a significant difference in age between the DHH and TH groups. To balance the age distribution for both DHH and TH children, we utilized the weighting method (Zou et al., 2019). A weight variable was used to balance the sample before analysis, and this works by assigning to each participant a different weighting ratio to reflect its relative importance to be taken into account during analysis (Zou et al., 2019). This method is most often seen in the analysis of survey data to readjust the sample to represent corresponding proportions in the population for variables such as age and gender (Royal, 2019; Zou et al., 2019).

## Results

### Group Differences

Table 2 shows the mean scores for the family system (family cohesion, family adaptability, and parental emotion communication) and children’s emotional functioning (positive and negative emotion expressions and emotion recognition). Independent samples *t*-tests revealed that the DHH group reported lower levels of family cohesion than the TH group, *t*(203) = 2.45, *p* = .015. No group differences were found for family adaptability, *t*(203) = 1.26, *p* = .209, or for parental emotion communication *t*(203) = −1.05, *p* = .296. As to children’s emotional functioning, DHH children were rated lower on positive emotion expression than TH children, *t*(203) = 4.32, *p* < .001, while no group differences were noted in negative emotion expression, *t*(203) = −1.25, *p* = .211, or in emotion recognition, *t*(203) = .28, *p* = .784. See Supplementary Appendix S2 for correlations between the study variables. See Supplementary Appendix S3 for correlations between the study variables and the family socioeconomic factors.

**Table 2:** Psychometric properties and mean scores (standard deviation) for all variables

	*N* items	Scale	Cronbach’s α	Mean (*SD*)		*t*-Value^a^
				DHH	TH	
Family cohesion	16	1–5	.91	3.4 (0.7)	3.6 (0.6)	2.45^*^
Family adaptability	14	1–5	.86	3.3 (0.6)	3.4 (0.5)	1.26
Parental emotion communication	10	0–4	.79	3.1 (0.4)	3.0 (0.5)	−1.05
Positive emotion expression	6	1–5	.72	3.2 (0.5)	3.5 (0.5)	4.32^**^
Negative emotion expression	8	1–5	.76	2.3 (0.6)	2.2 (0.5)	−1.25
Emotion recognition	6	1–5	.77	3.5 (0.6)	3.6 (0.6)	0.28

*Note.* Significance (2 tailed) ^**^p < .001, ^*^p < .05. DHH = deaf or hard-of-hearing; TH = typically hearing.

a Weighted and pooled results after multiple imputation.

### Moderating Effect of Hearing Status on the Relationship Between the Family System and Children’s Emotional Functioning

To examine the extent to which the relationships between the family system and children’s emotional functioning were moderated by hearing status, hierarchal regression analyses were conducted, respectively, with each component of children’s emotional functioning as the dependent variable. Due to multicollinearity of family cohesion with family adaptability (see Supplementary Appendix S2), we could not enter both variables in the regression model, and the decision was made to remove family adaptability from the analyses. In our study, the families with DHH children followed a 1-year training program at the Northern China early intervention center, which required children to attend classes at the early intervention center for 5 days a week and 8 hr per day. Consequently, these parents had less autonomy in their decision-making, setting their daily routines, and other relevant choices on a day-to-day basis during this year. In other words, family adaptability might have been subjected to the influence of the program set by the early intervention center. Additionally, we were unable to formulate a hypothesis for family adaptability in this study due to a lack of relevant literature.

**Figure 1:**
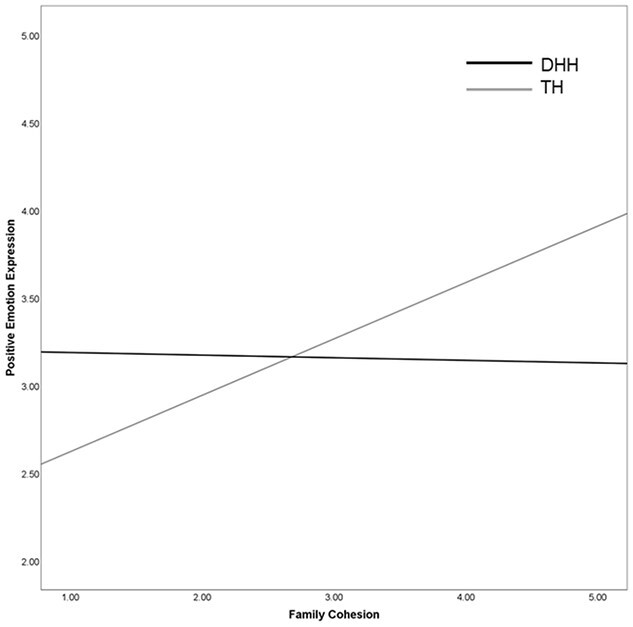
Interaction effect between family cohesion and positive emotion expression note: DHH = deaf or hard-of-hearing; TH = typically hearing.

Table 3 shows the outcomes of the regression models. In the analysis of children’s positive emotion expression, adding interactions of group with the family system variables improved the model fit (see Figure 1). For TH children, higher levels of family cohesion were related to more positive emotion expression. In DHH children, higher levels of family cohesion were unrelated to children’s positive emotion expression. No other effects were observed.

**Table 3:** Regression analyses between family system and children’s emotional functioning variables (weighted and pooled results)

	Positive emotion expression		Negative emotion expression		Emotion recognition	
	*b*	*p*	*b*	*p*	*b*	*p*
Step 1	*R^2^* = .11^**^		*R^2^* = .05^*^		*R^2^* = .01	
Intercept	2.89	**<.001**	1.94	**<.001**		
Group	−0.30	**<.001**	0.05	.518		
Family cohesion	0.11	.096	−0.13	**.046**		
Parental emotion communication	0.06	.479	0.25	**.003**		
Step 2	Δ*R^2^* = .12^**^		Δ*R^2^* = .04^*^		Δ*R^2^* = −.01	
Intercept	2.37	**<.001**	2.15	**<.001**		
Group	0.61	.263	−0.48	.365		
Family cohesion	0.34	**.001**	−0.06	.540		
Parental emotion communication	−0.04	.716	0.10	.396		
Family cohesion × group	−0.38	**.005**	−0.10	.461		
Parental emotion communication × group	0.14	.413	0.29	.087		

*Note*. Change in *R^2^*: *p < .05, **p < .001. In bold, *p* < .05. Group was coded as 0 = typically hearing; 1 = deaf or hard-of-hearing.

In the analysis of children’s negative emotion expression, adding interactions of group with the family system variables improved the model, although none of the interactions reached significance. In both groups, more parental emotion communication was related to more negative emotion expressions in children, whereas higher levels of family cohesion were related to less negative emotion expression. No other effects were noted.

In the analysis for children’s emotion recognition, adding interactions for group with the family system variables did not improve the model fit. No significant effects were observed.

In Supplementary Appendix S4, we report the regression model with family adaptability included and family cohesion removed.

## Discussion

This study aimed to examine possible relations of family functioning with DHH and TH children’s emotional functioning. In the current literature, there are no studies to the best of our knowledge that link family functioning (cohesion, adaptability, and parental emotion communication) with children’s emotional functioning in the DHH population even though the important role parents play for DHH children is repeatedly mentioned (Ketelaar et al., 2017; Koester & Lahti-Harper, 2010; Spahn et al., 2003). Outcomes indicated that families with a DHH child reported lower family cohesion than families with a TH child, whereas no group differences were shown for parental emotion communication or adaptability. Yet, higher levels of family cohesion was related not only to less negative emotion expression in both groups but also to more positive emotion expression in families with TH children only. Unexpectedly, more parental emotion communication was related to more negative emotion expressions in both DHH and TH children. No other relationships were observed. The implications of these findings will be explored in detail below.

As expected, higher levels of family cohesion were related to less negative emotion expression in both DHH and TD children. Note, however, that the scores for negative emotion expressions were relatively low for both DHH and TH groups in our study. This may be related to Eastern collectivistic cultures that prioritize group harmony and interpersonal relationships and tend to minimize negative emotion expressions as it can threaten group harmony (Friedlmeier et al., 2011; Suveg et al., 2014; Tao et al., 2012).

Yet, higher levels of family cohesion were also related to more positive emotion expressions, but only for TH children. Remarkably, levels of family cohesion and positive emotion expressions were both lower in DHH children than in TH children. These differences may be a result of how these families spend their time together. Hearing families with a DHH child in the current study are fully dedicated to the program at the early intervention center and their child’s learning, which also provides audiological checkups at the clinic for the children. This could have had a large impact on the cohesion levels in these families, as these families might have less time or possibilities for shared leisure activities together. Additionally, parents with a DHH child may spend a considerable amount of their time together on visits to the hospital, to the speech therapist or audiologist, and attending family counselling sessions, among other necessary duties. These activities allow families to share time together, but this time together are not necessarily positive events or promoting positive emotional experiences (Moskowitz et al., 2019; Robertson et al., 2007). This might explain why the levels of family cohesion were found unrelated to levels of positive emotion expressions for DHH children, as these children might indeed spend less time in fun or leisure activities with their family members.

Parental communication on emotions in the family system, on the other hand, was unexpectedly related to more negative emotion expressions in both groups of children. Possibly, open communication between parents and their children on their emotions may encourage the expression of emotions to flow more easily with the children. Especially with negative emotions, open expression within the family reduces suppression and subsequent dysregulation from overarousal, which can consequentially lead to psychopathology symptoms (Le & Impett, 2016; Panfile & Laible, 2012; Shaffer et al., 2012). Not only this, encouraging expression of negative emotions allows parents to model and teach their child how to manage these emotions and cope appropriately, which can largely improve the relationship quality (Le & Impett, 2016). Additionally, some parents of DHH children may be more protective (Eyuboglu et al., 2019), and these parents may be quick to react to any and every problem their children may have, whether it be physically or emotionally. Future studies could further explore this post hoc explanation and examine the assumptions formulated here.

Furthermore, unexpectedly, while the family system was related to children’s emotion expressions, it had no impact on children’s emotion recognition. Potentially, the skill to recognize basic emotions in children was too simple for these children, and the family may impact children less on simple skills such as this. On the other hand, emotion expressions function as signals to others and can reflect on an individual’s intentions and goals in the relationship (Scherer, 2000). Thus, emotion expression is communicative and is related to building and maintaining social relationships, which is vital in maintaining family systems (Olson, 2000).

### Limitations and Future Research

This study aimed to add to current knowledge on the relations between the family components and children’s emotional functioning for both DHH and TH children. Specifically, research on this relation for DHH children is equivocal and scarce. As the family plays an important role for young children, it is important to understand how this links to developments of emotional functioning, especially when families are adjusting to novel situations. Nevertheless, there are some limitations of this study that should also be mentioned.

First, most children have at least one CI and all have hearing parents, but the DHH population is also heterogeneous and thus individual differences might involve the degree of hearing loss or audiological interventions. The intensive family-centered early intervention programs that the DHH children in this study received may also contribute to the positive outcomes. Additionally, this study’s main focus was on DHH children with hearing parents, yet families with DHH parents may function differently to those with hearing parents as these DHH parents have a lifetime of experience, especially in adequate communication means with their children (Leigh et al., 2004). Further, Leigh found that children with either DHH or hearing parents did not differ significantly on whether they built a secure or insecure attachment style. Although it seems DHH children are more often found to present with a different attachment style than their parents; more likely, culture and education type plays a role here. Other subpopulations of the DHH group include children with additional disabilities, primary use of sign language, and in special or mainstream education. These subpopulations may all adapt to being DHH differently. These issues could be addressed in future studies in order to gain a deeper understanding of the individual differences within the group and its relationship with other areas of functioning.

Second, the cross-sectional nature of this study limits the conclusions for the directionality of the relations between the family system and children’s emotional functioning. Past research has shown that families function as a dynamic system that consistently changes over time (Cox & Paley, 2003; Olson, 2000), and children develop differently over the lifespan (Most & Michaelis, 2012). Moreover, as mentioned earlier, this study removed family adaptability from the regression model due to its high correlation with family cohesion. During the program, a stronger emotional bond between parent and child can protect them from stress and negative events, and this is where family cohesion and parental emotion communication may be vital in facilitating this process. A follow-up study that checks for changes in family functioning, specifically after the child has finished his or her year in the early intervention center, might shed light on the causality of the relationships assumed in this study as well as the effect of the 1-year program at the early intervention center.

Third, for future studies, examining the attachment between parents and their DHH child could inform us about the family system even before toddlerhood, which sets an important precedence (Thomson et al., 2011). Further, currently many studies have already looked at how attachment may influence emotional development in TH children, while there are no studies to our knowledge on DHH children.

Finally, cross-cultural comparisons could also be a direction for future research. Currently, limited literature is available on how families may function differently for DHH children, and subsequently on how it affects their social–emotional development for Eastern cultures, and there are even lesser studies that directly compare young DHH children between the West and the East.

## Conclusions

This study showed that the quality time family members spend together is linked to emotion expression for both DHH and TH children. As emotion expression is shaped within the culture and environment that one lives, children pick up many implicit rules through daily interactions with their family, for example, while overhearing arguments between parents or siblings. Although for DHH children, learning through interactions and observations could be more challenging indeed. Besides their hearing loss, they can easily miss certain information, for instance, when there is too much background noise or when there is more than one speaker, such as at the dinner table with all their family present. Moreover, the content of DHH children’s daily activities with their parents might be different; hospital visits instead of visits to the zoo, or speech therapy instead of going to the swimming pool or the playground. Our findings highlight the importance of a family environment that is attuned to the children’s needs and open to communication, where it is equally important to create opportunities to have fun together. Therefore, family-centered early intervention programs may contribute to DHH children’s family functioning and facilitate their emotional development, such as that parents who are really involved in DHH children’s early intervention programs usually are able to communicate better with their children than those parents who are not (Sass-Lehrer et al., 2016). Priority should also be placed on increasing awareness for doctors and counselors on specific interactions within families, such as stress after diagnosis that some parents may encounter (e.g., Calderon & Greenberg, 2011; Luckner & Velaski, 2004). Furthermore, families that have a DHH child may face stigma in the community, so providing resources that assist in building access to social support also seems vital for healthy family functioning. Studies like this might also inform professionals working with DHH children on how to connect to the parents and children, such as including leisure activities for the family within the early intervention program itself.

## Supplementary Material

CN_DHH_Family_appendix_enab044
