# Supplementary material for: Family Systems and Emotional Functioning in Deaf or Hard-of-Hearing Preschool Children
Source: J Deaf Stud Deaf Educ. 2022 Jan 31;27(2):125–36. doi: 10.1093/deafed/enab044 (PMC8929678; doi:10.1093/deafed/enab044)
Supplement: CN_DHH_Family_appendix_enab044 [file cn_dhh_family_appendix_enab044.docx]

**Appendices**

**Appendix 1**

*Overview of Missing Data*

|  | **DHH** |  |  | **TH** |  |  |
| --- | --- | --- | --- | --- | --- | --- |
|  | ***n*** | **Missing count** | **%** | ***n*** | **Missing count** | **%** |
| Age | 106 | 0 | 0 | 99 | 0 | 0 |
| Gender | 106 | 0 | 0 | 99 | 0 | 0 |
| Non-verbal intelligence score | 75 | 31 | 29.2 | 90 | 9 | 9.1 |
| Maternal education level | 104 | 2 | 1.9 | 70 | 29 | 29.3 |
| Paternal education level | 103 | 3 | 2.8 | 69 | 30 | 30.3 |
| Income | 84 | 22 | 20.8 | 54 | 45 | 45.5 |
| Family cohesion | 105 | 1 | .9 | 94 | 5 | 5.1 |
| Family adaptability | 105 | 1 | .9 | 93 | 6 | 6.1 |
| Parent-child communication | 105 | 1 | .9 | 95 | 4 | 4.0 |
| Positive emotion expression | 106 | 0 | 0 | 99 | 0 | 0 |
| Negative emotion expression | 106 | 0 | 0 | 99 | 0 | 0 |
| Emotion recognition | 106 | 0 | 0 | 99 | 0 | 0 |

*Note.* DHH = deaf or hard-of-hearing; TH = typically hearing

**Appendix 2**

*Pearson Correlations Between Family System and Emotional Functioning (Weighted and Pooled Results)*

|  | Correlation coefficient for all children (for DHH / TH children) | | | | | |
| --- | --- | --- | --- | --- | --- | --- |
|  | 1. | 2. | 3. | 4. Positive emotion expression | 5. Negative emotion expression | 6. Emotion Recognition |
| 1. Family cohesion | - | - | - | .19 (-.02/.34*) ^a^ | -.08 (-.09/-.03) | *.*06 (-.01/.15) |
| 2. Family adaptability | .84 (.88**/.77**) ^a^ | - | - | .11 (-.02/.22*) | .02 (.02/.04) | .10 (.02/.21*) |
| 3. Parental emotion communication | .37 (.31**/.48**) | .41 (.41**/.44**) | - | .08 (.07/.13) | .17 (.25*/.07) | .05 (-.05/.16) |

*Note*. DHH = deaf or hard-of-hearing; TH = typically hearing

^a^ Significance of the z-score after Fisher’s r-to-z transformation, which was applied to compare the strength of correlations.

***p*<.001 **p*<.05 for correlations between family and emotion variables.

**Appendix 3**

*Pearson Correlations Between Socioeconomic Factors and All Study Variables (Weighted and Pooled Results)*

|  | (for DHH / TH children) | | |
| --- | --- | --- | --- |
|  | Maternal education | Paternal education | Household income |
| 1. Family cohesion | -.11/.13 | -.09/.10 | .12/.09 |
| 2. Family adaptability | -.06/.17 | -.01/.03 | .20*/.21* |
| 3. Parental emotion communication | -.02/.10 | .02/.003 | .07/-.03 |
| 4. Positive emotion expression | .15/.18 | .08/.12 | .01/.06 |
| 5. Negative emotion expression | .10/-.11 | .09/-.10 | .08/.12 |
| 6. Emotion recognition | .17/.06 | .05/-.02 | -.01/.004 |

*Note.* DHH = deaf or hard-of-hearing; TH = typically hearing.

***p*<.001 **p*<.05 for correlations between variables

**Appendix 4**

*Regression Analyses Between Family System (with Family Adaptability Instead of Family Cohesion) and Children’s Emotional Functioning Variables (Weighted and Pooled Results)*

|  | Positive emotion expression | | Negative emotion expression | | Emotion recognition | |
| --- | --- | --- | --- | --- | --- | --- |
|  | *b* | *p* | *b* | *p* | *b* | *p* |
| **Step 1** | *R^2^* = .10** | | *R^2^* = .04 | | *R^2^* = .01 | |
| Intercept | 3.02 | **<.001** |  |  |  | |
| Group | -.32 | **<.001** |  |  |  | |
| Family adaptability | .05 | .504 |  |  |  | |
| Parental emotion communication | .09 | .295 |  |  |  | |
| **Step 2** | Δ*R^2^* = .10** | | Δ*R^2^* = .03 | | Δ*R^2^* = .00 | |
| Intercept | 2.53 | **<.001** |  |  |  | |
| Group | .47 | .422 |  |  |  | |
| Family adaptability | .23 | .066 |  |  |  | |
| Parental emotion communication | .05 | .673 |  |  |  | |
| Family adaptability x group | -.29 | .074 |  |  |  | |
| Parental emotion communication x group | .06 | .742 |  |  |  | |

*Note*. Change in *R^2^*: **p*<.05, ***p*<.001.

In bold *p*<.05.

Group was coded as 0 = Typically hearing 1= deaf or hard-of-hearing.
